# Editorial: Biological-Inspired Autonomous Mobile Manipulation: Challenges in Design, Control, and Real-World Applications

**DOI:** 10.3389/fnbot.2022.928465

**Published:** 2022-06-15

**Authors:** Zhenshan Bing, Fei Chen, Rui Li, Junpei Zhong, Qiang Li

**Affiliations:** ^1^Department of Informatics, Technical University of Munich, Munich, Germany; ^2^Department of Mechanical and Automation Engineering, The Chinese University of Hong Kong, Hong Kong, Hong Kong SAR, China; ^3^School of Automation, Chongqing University, Chongqing, China; ^4^Department of Rehabilitation Sciences, Hong Kong Polytechnic University, Hong Kong, Hong Kong SAR, China; ^5^Center for Cognitive Interaction Technology (CITEC), Bielefeld University, Bielefeld, Germany

**Keywords:** autonomous mobile manipulation, biological-inspired mechanism design, biological-inspired control, neuromorphic sensing and computation, brain-inspired navigation

This special issue on “*Biological-Inspired Autonomous Mobile Manipulation: Challenges in Design, Control, and Real-World Applications*” of Frontier in Neurorobotics introduces the latest research and advances in theoretical and experimental results dealing with biological-inspired and conventional techniques for the design, control, and real-world applications of autonomous mobile manipulation systems.

Research in autonomous mobile manipulation aims to develop mobile robotic systems with manipulation capabilities that enable them to perform complex tasks in dynamic, unstructured, and field environments, for which task-tailored design, control, and novel application methods are required. Some widely used applications include but are not limited to, logistics, industrial maintenance, remote medical examination, and service robotic tasks. For this purpose, autonomous mobile manipulation systems should be able to perform and coordinate different skills, such as locomotion, perception, manipulation, and grasping. To acquire more advanced autonomy and operate within the real world, such systems should be further investigated with the following capacities: (1) they should have generality and adaptability across unseen tasks; (2) perception of their environments via sensors that typically deliver high-dimensional data with low response latency and energy efficiency; (3) they should be capable of traveling in challenging scenarios and performing complex tasks with certainty; (4) they should have system stability and the complexity to integrate many hardware components as well as software algorithms for different functionalities.

In nature, biological intelligence has great capabilities and generalities to locomote, perceive, and act in the real world exceptionally well, outperforming state-of-the-art robots in almost every aspect of life. An increasing number of works are investigating biologically-inspired robotic studies in many areas, ranging from the biomimetic mechanical design of robots, neuromorphic sensing, and computing to brain-inspired navigation. This Research Topic seeks to invite theoretical and experimental results dealing with biological-inspired and conventional techniques for the design, control, and real-world applications of autonomous mobile manipulation systems. Specifically, this Research Topic investigates interdisciplinary innovation in many areas of robots, such as information perception, novel control architectures, biomimetic mechanism, motion planning, grasping and manipulation, human-robot interaction, and artificial intelligence and machine learning.

After careful peer reviews and revision, four representative papers were accepted for publication in this special issue. These papers represent four important application areas of brain-inspired algorithms. The related summary of every topic is given below. We strongly recommend reading the entire paper if interested.

## Topic 1: A Survey of Multifingered Robotic Manipulation: Biological Results, Structural Evolvements and Learning Methods

Dexterous manipulation is a “big” challenge in the robotic domain. From the neuro-inspired point of view, this paper did a survey on the human hand's dexterity from physiological aspects, summarized the task-oriented robotic hands and their sensing and motor system, and finally introduced the state of the art in learning and control approaches of multi-fingered robotic hands. In the last section, the authors report on three challenges for learning and the control of multi-fingered hands compared with articulated manipulators or other robots: (1) high dimensions in state and action space (2) limited and constrained workspace, complex tasks and frequently switching control mode (3) difficulties in obtaining good training data from teachers. Finally, future work was proposed not only from a single aspect but also from comprehensive consideration to improve robotic hand dexterity from neuro-designing-learning and controlling.

## Topic 2: Event-Based Circular Detection for AUV Docking Based on Spiking Neural Network

The human brain is an advanced computation machine with extremely high energy efficiency (10^∧^11 neurons, 10^∧^15 synapse for 16.6 W). The spiking neural network (SNN) is viewed as an important advancement in constructing a more intelligent system with fewer computation resources. Zhang et al. present their latest discovery on implementing an SNN based perception system for underwater robotics. They used an SNN combined with a dynamic vision sensor (DVS) to efficiently detect lighting landmarks, thus enabling the robot to dock with other components in a robust and energy-saving manner.

## Topic 3: A Bionic Spatial Cognition Model and Method For Robots based On Hippocampus Mechanism

The hippocampus and the entorhinal cortex, which it surrounds, act as navigational functions in the brain. These cortexes contain “place cells,” which aid animals in navigating mazes, and “grid cells,” which serve as the brain's internal GPS system and other cells within the functional spatial system in the brain. Such anatomical evidence inspired Yuan et al. to build five distinct cell models in the spatial cells model. Functionally, it adopts the velocity and direction of the ego-movement. The physiological firing characteristic of the cells is also stimulated by using the embodied robot inputs. This characteristic provides a number of benefits over formal neural networks, including the capacity to simulate dynamical network activities and computation in real-time. Its physiological feasibility also accounts for the anti-interference ability and robustness in noise, which can be applied to some of the biological-inspired robot control methods that we introduce in this special issue.

## Topic 4: Creating Better Collision-Free Trajectory for Robot Motion Planning by Linearly Constrained Quadratic Programming

Due to the randomness of sampling, many sampling-based motion planning algorithms can only compute collision-free trajectories with poor local optimization. Liu et al. presented their new trajectory optimization technique based on linearly constrained quadratic programming. The algorithm can remove redundant motions in the parameter space of trajectory and convert collision avoidance conditions to linear constraints to ensure the absolute safety of trajectories. Their results successfully demonstrate the feasibility and effectiveness of the proposed method compared with other trajectory optimization methods.

In summary, the papers selected for this special issue show the significant effect and application potential of biologically-inspired algorithms. There are still many challenges in this field that require future research attention, such as more biologically plausible methods and well-developed applications.

## Author Contributions

All authors listed have made a substantial, direct, and intellectual contribution to the work and approved it for publication.

## Conflict of Interest

The authors declare that the research was conducted in the absence of any commercial or financial relationships that could be construed as a potential conflict of interest.

## Publisher's Note

All claims expressed in this article are solely those of the authors and do not necessarily represent those of their affiliated organizations, or those of the publisher, the editors and the reviewers. Any product that may be evaluated in this article, or claim that may be made by its manufacturer, is not guaranteed or endorsed by the publisher.

